# Deubiquitinase function of A20 maintains and repairs endothelial barrier after lung vascular injury

**DOI:** 10.1038/s41420-018-0056-3

**Published:** 2018-05-16

**Authors:** Dheeraj Soni, Dong-Mei Wang, Sushil C. Regmi, Manish Mittal, Stephen M. Vogel, Dirk Schlüter, Chinnaswamy Tiruppathi

**Affiliations:** 1 Department of Pharmacology and Center for Lung and Vascular Biology, College of Medicine, University of Illinois, Chicago, IL USA; 2 Institute of Medical Microbiology and Hospital Hygiene, Otto-von-Guericke University Magdeburg, Magdeburg, Germany

## Abstract

Vascular endothelial cadherin (VE-cad) expression at endothelial adherens junctions (AJs) regulates vascular homeostasis. Here we show that endothelial A20 is required for VE-cad expression at AJs to maintain and repair the injured endothelial barrier. In endothelial cell (EC)-restricted *Tnfaip3* (A20) knockout (*A20*^*∆EC*^) mice, LPS challenge caused uncontrolled lung vascular leak and persistent sequestration of polymorphonuclear neutrophil (PMNs). Importantly, *A20*^*∆EC*^ mice exhibited drastically reduced VE-cad expression in lungs compared with wild-type counterparts. Endothelial expression of wild-type A20 but not the deubiquitinase-inactive A20 mutant (A20^C103A^) prevented VE-cad ubiquitination, restored VE-cad expression, and suppressed lung vascular leak in *A20*^*∆EC*^ mice. Interestingly, IRAK-M-mediated nuclear factor-κB (NF-κB) signaling downstream of TLR4 was required for A20 expression in ECs. interleukin-1 receptor-associated kinase M (IRAK-M) knockdown suppressed basal and LPS-induced A20 expression in ECs. Further, in vivo silencing of IRAK-M in mouse lung vascular ECs through the CRISPR-Cas9 system prevented expression of A20 and VE-cad while augmenting lung vascular leak. These results suggest that targeting of endothelial A20 is a potential therapeutic strategy to restore endothelial barrier integrity in the setting of acute lung injury.

## Introduction

Expression of vascular endothelial cadherin (VE-cad) at endothelial adherens junctions (AJs) is vital for endothelial barrier integrity^1,2^. Loss of VE-cad expression at endothelial AJs induces persistently increased vascular permeability and uncontrolled extravasation of leukocytes into lungs leading to inflammatory lung injury^3–5^. VE-cad forms Ca^2+^-dependent homophilic *cis* and *trans* dimers at inter-endothelial AJs to establish cell-to-cell adhesion^2–6^. The cytoplasmic domain of VE-cad interacts with several intracellular proteins, including β-catenin, plakoglobin, and p120-catenin^2–6^. VE-cad phosphorylation at Y-658, Y-685, and Y-731 induces disassembly of endothelial AJs that results in increased vascular permeability and PMN extravasation into lungs^7–11^. The transmembrane protein, VE protein tyrosine phosphatase (VE-PTP), exclusively expressed in endothelial cells (ECs), interacts with and constitutively dephosphorylates VE-cad to maintain endothelial barrier integrity^10,12,13^. Recent knock-in mouse models have revealed that VE-cad phosphorylation at Y-685 promotes increased vascular permeability whereas VE-cad phosphorylation at Y-731 induces PMN extravasation^14^. Challenge with infectious Gram-negative bacterial cell wall component lipopolysaccharide (LPS) promotes loss of VE-cad at endothelial AJs to trigger acute lung injury (ALI) characterized by excessive extravasation of inflammatory cells into lung tissue, increased lung vascular permeability, and accumulation of protein-rich edema^15–18^. However, how VE-cad expression is basally regulated and how LPS-induced loss of VE-cad is restored after inflammatory injury are poorly understood.

A20/TNFAIP3, a ubiquitin-editing enzyme, is an endogenous negative master regulator of the NF-κB signal transduction pathway^19,20^. A20 is a cytoplasmic protein containing an N-terminal ovarian tumor (OTU) domain and seven zinc-finger domains in its C-terminal. The OTU domain possesses deubiquitinase (DUB) activity, which mediates de-ubiquitination of K63-linked ubiquitin chains in proteins and the C-terminal fourth zinc-finger domain possesses ubiquitin E3 ligase activity, which causes K48-linked ubiquitination of target proteins for proteasomal degradation^19–21^. A20’s C-terminal fourth zinc-finger domain is essential for A20 binding to ubiquitin and function^22^. In vitro studies have shown that A20 can cleave both that K63- and K48-linked polyubiquitin chains from proteins^22–24^ and also A20 can block E2–E3 interaction to prevent ubiquitination^25^. A20 is an inducible NF-κB response gene and thereby plays a critical role in the negative feedback regulation of NF-κB and mitogen-activated protein kinase (MAPK) signaling pathways to restrict inflammatory responses^19,20,26–29^. A20-deficient mice were born at normal Mendelian ratios but died prematurely due to multi-organ inflammation^27^. In the human case, genome-wide association studies showed that polymorphisms in the A20 gene were associated with a number of inflammatory diseases^19^. Importantly, it has been reported that A20’s DUB activity is essential for the inhibition of apoptosis, NLRP3 inflammasome activation, and necroptosis^27,30,31^. We showed recently that endotoxin induced PMN sequestration in lungs and lung vascular leak were markedly reduced in A20 overexpressing (*Kcnip3* deficient) mice^29^. However, it is unclear whether A20 controls lung PMN sequestration and vascular leak by stabilizing VE-cad at endothelial AJs. As VE-cad phosphorylation-dependent ubiquitination may promote degradation of VE-cad to induce endothelial barrier dysfunction^9^, we surmised that A20’s DUB activity may stabilize the endothelial barrier by regulating the expression of VE-cad at endothelial AJs. We observed markedly reduced VE-cad expression in ECs of EC-restricted A20 knockout (*A20*^*∆EC*^) mice, and importantly *A20*^*∆EC*^ mice failed to restore the endothelial barrier after endotoxin challenge, indicating that A20 signaling in ECs is vital for the maintenance and repair of the endothelial barrier after ALI.

## Results

### EC-restricted A20 deletion in mice amplifies LPS-induced lung vascular inflammatory responses

**Fig. 1:**
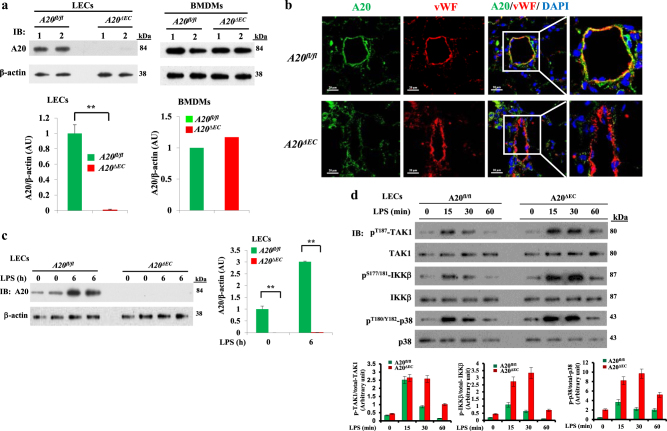
A20 deficiency augments LPS-induced signaling in endothelial cells. **a** A20 expression was determined by immunoblot (IB) analysis in lung endothelial cells (LECs) and bone marrow-derived macrophages (BMDMs) from *A20*^*fl/fl*^ and *A20*^*∆EC*^ mice. **p < 0.001, four separate cell preparations. **b** Immunostaining of lung sections from *A20*^*fl/fl*^ and *A20*^*∆EC*^ mice with antibody specific to A20 and endothelial marker vWF. **c** LECs from *A20*^*fl/fl*^ and *A20*^*∆EC*^ mice treated with LPS (1 µg/ml) were used for immunoblot to determine A20 protein expression. **p < 0.001, *n* = 4 experiments. **d** LECs from *A20*^*fl/fl*^ and *A20*^*∆EC*^ mice treated with LPS (1 µg/ml) for different time intervals were used for immunoblot analysis to determine phosphorylation of TAK1, IKKβ, and p38. Representative data from three experiments are shown in **a**-**d**

We created EC-restricted A20 (encoded by *Tnfaip3*) knockout (*A20*^*ΔEC*^) mice by crossing loxP-flanked A20 (*A20*^*fl/fl*^) mice^32^ with Cdh5-Cre-recombinase-expressing mice^11^. We observed complete loss of A20 expression in lung ECs (LECs) from *A20*^*ΔEC*^ mice compared with *A20*^*fl/fl*^ mice (Figs. 1a, b). A20 expression in myeloid cells (bone marrow-derived macrophages (BMDMs)) did not differ in *A20*^*ΔEC*^ and *A20*^*fl/fl*^ mice (Fig. 1a) suggesting that Cdh5-Cre specifically deletes A20 expression in ECs. Next, we measured LPS-induced A20 expression in LECs from *A20*^*fl/fl*^ (WT) and *A20*^*ΔEC*^ mice and observed that LPS-induced A20 expression in WT-LECs but not in *A20*^*ΔEC*^-LECs (Fig. 1c) further suggesting loss of A20 expression in LECs of *A20*^*ΔEC*^ mice. We also measured LPS-induced activation of transforming growth factor β-activated kinase 1 (TAK1), IκB kinase β (IKKβ), and p38 in LECs of *A20*^*fl/fl*^ and *A20*^*ΔEC*^ mice. As expected, LPS-induced phosphorylation of TAK1, IKKβ, and p38 was augmented in LECs of *A20*^*ΔEC*^ mice compared with *A20*^*fl/fl*^ (Fig. 1d). These findings demonstrate that A20 deficiency augments LPS-induced inflammatory signaling in LECs.

**Fig. 2:**
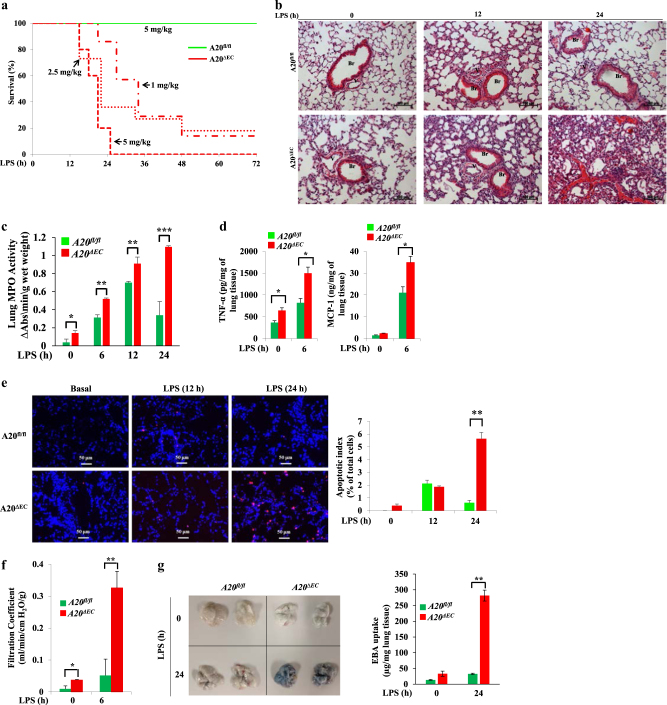
*A20*^*∆EC*^ mice exhibit heightened sensitivity to LPS-induced septic shock. **a** Survival of age- and weight-matched *A20*^*fl/fl*^ and *A20*^*∆EC*^ mice after administration of indicated LPS doses (mg/kg, i.p.). (*n* = 10 per genotype per LPS dose). **b** Hematoxylin-and-eosin staining of lung sections from *A20*^*fl/fl*^ and *A20*^*∆EC*^ mice challenged with LPS (2.5 mg/kg; i.p.) for different time points. Scale bars, 100 μm. *Br*bronchi, *V* vessel. **c** Myeloperoxidase (MPO) activity in lung tissue from *A20*^*fl/fl*^ and *A20*^*∆EC*^ mice challenged with LPS (2.5 mg/kg, i.p) for 0, 6, 12, and 24 h. *n* = 5 per genotype per time point. *p < 0.01; **p < 0.001; ***p < 0.0001; *A20*^*fl/fl*^ versus *A20*^*∆EC*^ mice. **d** Concentrations of cytokines TNF-α and MCP-1 in lungs of *A20*^*fl/fl*^ and *A20*^*∆EC*^ mice challenged with LPS (5 mg/kg, i.p) for 0 and 6 h. *n* = 5 per genotype. *p < 0.01, *A20*^*fl/fl*^ versus *A20*^*∆EC*^ mice. **e** TUNEL staining of lung tissue sections from *A20*^*fl/fl*^ and *A20*^*∆EC*^ mice shows apoptotic cells in *A20*^*∆EC*^ mice. Representative data from three experiments are shown. **p < 0.001, *A20*^*fl/fl*^ versus *A20*^*∆EC*^ mice. **f** Pulmonary microvessel liquid filtration coefficient in lungs of *A20*^*fl/fl*^ and *A20*^*∆EC*^ mice after LPS challenge (5 mg/kg, i.p.). *n* = 5 per genotype per time point. *p < 0.01; **p < 0.001; *A20*^*fl/fl*^ versus *A20*^*∆EC*^ mice. **g** *A20*^*fl/fl*^ and *A20*^*∆EC*^ mice challenged with LPS (5 mg/kg, i.p.) for 0 and 24 h were used to assess in vivo lung vascular leak by measuring Evans blue dye-conjugated with albumin (EBA) uptake. Left, representative photographs of the lungs are shown; right, quantified results are shown; *n* = 4 per genotype per time point. **p < 0.001; *A20*^*fl/fl*^ versus *A20*^*∆EC*^ mice

We examined the effect of A20 deletion in ECs on the susceptibility to endotoxin-induced septic shock. We observed that an LPS dose of 5 mg/kg produced no lethality in WT mice; in contrast 100% mortality was observed within 24 h of LPS challenge in *A20*^*ΔEC*^ mice (Fig. 2a). We also administered low doses of LPS (2.5 mg/kg; 1 mg/kg) to *A20*^*ΔEC*^ mice and observed ~80% mortality within 48 h of LPS challenge (Fig. 2a). Next, we examined pathological changes in lungs of *A20*^*fl/fl*^ and *A20*^*ΔEC*^ mice after challenging with LPS. Hematoxylin-and-eosin staining of lung sections from wild-type (WT) mice treated with LPS at a dose of 2.5 mg/kg showed perivascular infiltration of leukocytes at 12 h (Fig. 2b) and restoration to the basal level at 24 h (Fig. 2b). In contrast to WT mice, hematoxylin-and-eosin staining of lung sections from *A20*^*ΔEC*^ mice showed persistent infiltration of leukocytes and hemorrhage after LPS (2.5 mg/kg) challenge (Fig. 2b). Next, we determined the time course of LPS-induced sequestration of PMNs in the lungs of both mouse strains. Interestingly, we observed that LPS challenge caused persistent PMN sequestration in lungs of *A20*^*ΔEC*^ mice (Fig. 2c). We also observed significantly increased concentrations of tumor necrosis factor (TNF)-α and monocyte chemoattractant protein-1 (MCP-1) in the lungs of *A20*^*ΔEC*^ mice compared with WT (Fig. 2d). As observed in acute phase of ALI in humans^16^, LPS induces endothelial apoptosis and increased endothelial permeability to promote lung injury^15,17^. Therefore, we determined these responses in lungs of WT and *A20*^*ΔEC*^ mice. We observed markedly increased terminal deoxynucleotidyl transferase dUTP nick end labeling (TUNEL)-positive vascular cells in lungs of *A20*^*ΔEC*^ mice compared with WT mice (Fig. 2e). This finding is in agreement with the concept that A20 functions to prevent cell death that could occur due to apoptosis and necroptosis^33–35^. To study the effect of A20 deficiency in ECs on LPS-induced lung vascular permeability, we first measured the pulmonary vascular liquid filtration coefficient (*K*_*f,c*_) using isolated lung preparations of both genotypes. LPS challenge caused several-fold increases in liquid filtration coefficient in the lungs of *A20*^*ΔEC*^ mice compared with the WT counterparts (Fig. 2f). Next, we determined Evans blue dye-conjugated albumin (EBA) uptake in lungs to assess in vivo lung vascular leak. We observed that an LPS dose of 2.5 mg/kg failed to induce vascular leak in WT mice (Fig. 2g), whereas an LPS dose of 2.5 mg/kg for 24 h caused several-fold increases in EBA uptake over basal in lungs of *A20*^*ΔEC*^ mice (Fig. 2g), indicating uncontrolled lung vascular leakage in response to LPS in *A20*^*ΔEC*^ mice.

### A20 signaling in ECs is required for stabilizing VE-cad expression at AJs to maintain endothelial barrier integrity

**Fig. 3:**
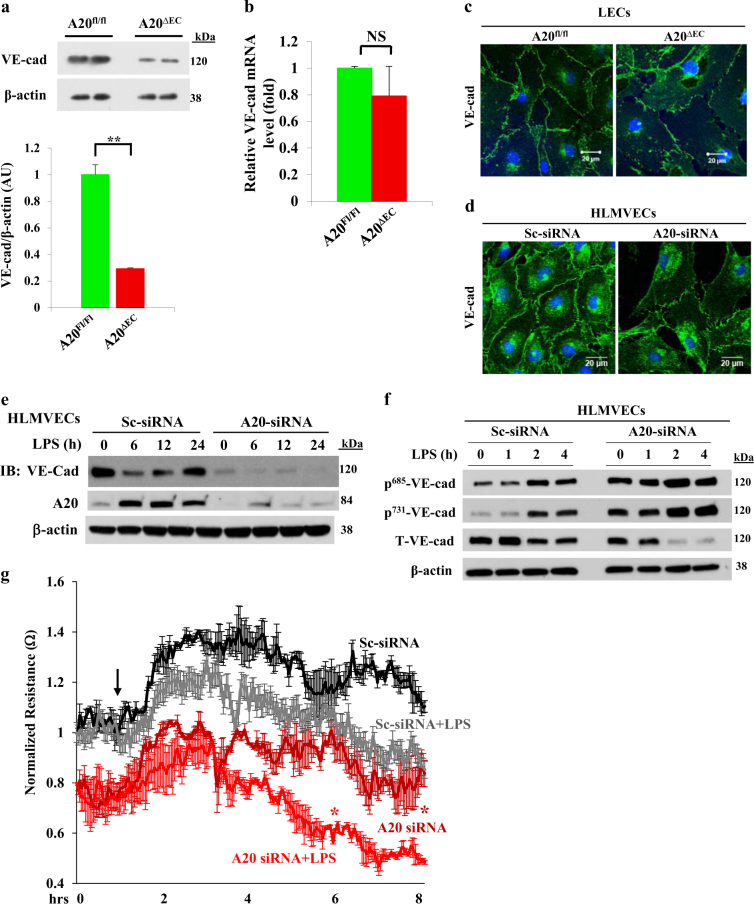
A20 deficiency in endothelial cells decreases VE-cad expression at AJs to impair endothelial barrier integrity. **a** Immunoblot analysis of VE-cad in lungs of *A20*^*fl/fl*^ and *A20*^*∆EC*^ mice. *n* = 4, in each group; **p < 0.001. **b** Quantitative RT-PCR analysis of VE-cad mRNA in lungs of *A20*^*fl/fl*^ and *A20*^*∆EC*^ mice. *n* = 4, in each group. **c** LECs from *A20*^*fl/fl*^ and *A20*^*∆EC*^ mice grown on coverslips were stained with VE-cad pAb (green) and analyzed by confocal microscopy. **d** HLMVECs were transfected with either scrambled-siRNA (Sc-siRNA) or A20- siRNA (100 nM). At 72 h after transfection, cells were challenged with LPS (1 µg/ml) for indicated times. The cell lysates were then used to measure LPS-induced A20 and VE-cad expression by IB. **e** HLMVECs transfected with either Sc-siRNA or A20- siRNA (100 nM) were immunostained with VE-cad pAb (green), and analyzed as in **b**. **f** HLMVECs were transfected as in **d** after pretreatment with proteasomal inhibitor MG132 (10 µM) for 2 h, challenged with LPS (1 µg/ml) for indicated time intervals were then used to determine VE-cad phosphorylation using antibody specific to phospho-Y^685^ and phospho-Y^731^. **c-f** Representative data from three experiments are shown. **g** HLMVECs transfected with Sc-siRNA or A20- siRNA as in **e**. At 24 h after transfection, cells plated on gold electrodes were challenged with LPS (1 µg/ml) in the presence of medium containing 10% FBS, and endothelial AJ integrity was monitored by TER (*n* = 4 per group). *p < 0.01, compared with respective controls

VE-cad is exclusively expressed in ECs and it functions at AJs to limit inflammatory cell extravasation and vascular leak^2^. Therefore, we determined VE-cad expression in the lungs of WT and *A20*^*ΔEC*^ mice. Surprisingly, we observed markedly reduced expression of VE-cad in lungs of *A20*^*ΔEC*^ mice compared with WT mice (Fig. 3a). However, we did not observe any significant alterations in the expression of VE-cad mRNA in *A20*^*ΔEC*^ mice compared with WT (Fig. 3b). To address whether the A20 expression in ECs is required for VE-cad expression at AJs, we stained LECs from WT and *A20*^*ΔEC*^ mice with anti-VE-cad Ab. We observed normal VE-cad expression at AJs in LECs of WT mice, whereas LECs from *A20*^*ΔEC*^ mice failed to form normal endothelial AJs (Fig. 3c). We also observed that knockdown of A20 using small interfering RNA (siRNA) resulted in markedly reduced VE-cad expression at AJs in human lung microvascular ECs (HLMVECs) (Fig. 3d). Next, we determined whether A20 expression in ECs is required for the restoration of the endothelial barrier after LPS-induced disassembly of endothelial AJs. LPS challenge resulted in a time-dependent decrease in VE-cad expression in HLMVECs and its expression was restored to basal within 24 h of LPS challenge (Fig. 3e). In contrast, after A20 knockdown in HLMVECs, LPS challenge resulted in loss of VE-cad, and restoration of VE-cad expression was blocked (Fig. 3e). As VE-cad phosphorylation allows PMN transmigration and permeability increase across the endothelial barrier, we observed that basal, as well as LPS-induced VE-cad phosphorylation on Y-685 and Y731 was augmented in A20 knockdown HLMVECs compared with the control (Fig. 3f). Next, we determined endothelial barrier integrity by measuring changes in transendothelial monolayer electrical resistance (TER) in HLMVECs under control conditions and after A20-knockdown. We observed that A20 knockdown in HLMVECs caused a significantly greater LPS effect on TER than the control (Sc-siRNA treated) (Fig. 3g). These results taken together indicate that EC-A20 plays a critical role in regulating endothelial barrier stability.

### A20 inhibits ubiquitination of VE-cad to maintain and restore endothelial AJ integrity after LPS-induced endothelial injury

**Fig. 4:**
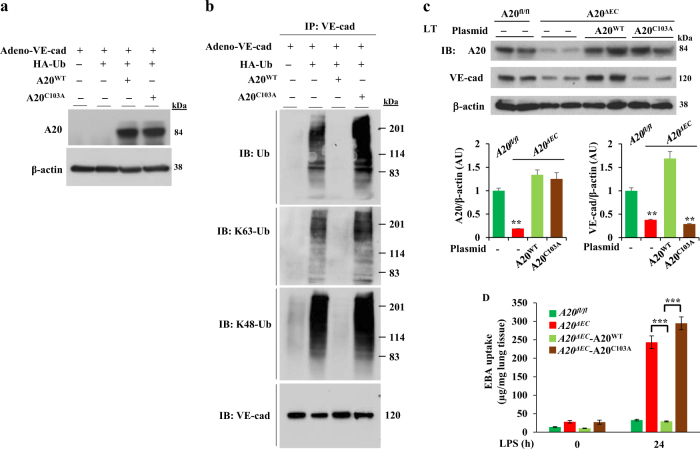
DUB activity of endothelial A20 restores endothelial AJs integrity after LPS-induced endothelial injury. **a** HMECs were co-transfected with an HA-tagged ubiquitin-expressing plasmid and with either A20^WT^ or A20^C103A^ plasmid. At 24 h after transfection, cells were infected with recombinant adenovirus-expressing VE-cad (Adeno-VE-cad). At 72 h, cell lysates were used for IB to determine A20 expression. Prior to lysis, cells were pretreated with MG132 (10 µM) for 2 h. **b**, As in **a**, lysates were immunoprecipitated (IP-ed) using VE-cad antibody. The IP-ed samples were used for IB analysis to determine ubiquitination of VE-cad. Top panel, blotted with pan-ubiquitin-specific antibody; Second panel, blotted with ubiquitin K63-linkage-specific antibody; Third panel, blotted with ubiquitin K48-linkage-specific antibody; Bottom panel, blotted with VE-cad-specific antibody. Representative data from three experiments are shown. **c** *A20*^*∆EC*^ mice were injected i.v. with liposome-A20 plasmid complexes containing either wild-type A20 (A20^WT^) or DUB-inactive A20-C103A mutant (A20^C103A^). At 96 h after injection, lungs harvested were used to determine A20 and VE-cad expression by IB. *n* = 4 mice each group; **p < 0.001, A20 expression-*A20*^*∆EC*^ vs A20^WT^ or A20^C103A^; **p < 0.001, VE-cad expression: *A20*^*fl/fl*^ vs *A20*^*∆EC*^, *A20*^*∆EC*^ injected with A20^WT^ vs *A20*^*∆EC*^, *A20*^*∆EC*^ injected with A20^WT^ vs *A20*^*∆EC*^ injected with A20^C103A^. **d** As in **c** *A20*^*fl/fl*^*, A20*^*∆EC*^, *A20*^*∆EC*^ injected with A20^WT^ construct, and *A20*^*∆EC*^ injected with A20^C103A^ construct were challenged with LPS (2.5 mg/kg) for 24 h and then in vivo lung vascular leak was determined. *n* = 4 mice per group; ***p < 0.0001; *A20*^*∆EC*^ mice versus *A20*^*∆EC*^ mice injected with A20^WT^ construct; *A20*^*∆EC*^ mice injected with A20^WT^ construct versus *A20*^*∆EC*^ mice injected with A20^C103A^ construct

As we observed that A20 deficiency promotes a decrease in VE-cad expression to destabilize the endothelial barrier, we tested whether A20 prevents ubiquitination of VE-cad and in effect sequesters VE-cad to AJs to stabilize the endothelial barrier. We co-expressed HA-tagged ubiquitin with either WT-A20 (A20^WT^) or DUB-inactive mutant A20 (A20^C103A^) in human dermal microvascular EC line (HMEC). We used HMEC for this experiment because HMECs have a higher transfection efficiency than primary ECs^36^. At 24 h after transfection, cells were infected with a recombinant adenovirus-expressing VE-cad (Adeno-VE-cad). At 72 h, cells were used to assess VE-cad ubiquitination. We observed VE-cad containing both K63- and K48-linked polyubiquitin chains (Figs. 4a, b). Interestingly, expression of WT-A20 but not the DUB-inactive mutant A20 prevented VE-cad ubiquitination (Fig. 4b), indicating that A20 might be cleaving both the K63- and K48-linked polyubiquitin chains from VE-cad^22–24^ or preventing ubiquitination of VE-cad to restore the endothelial AJs^25^. To address the in vivo relevance of A20 in regulating the endothelial barrier, we used a liposome-mediated gene delivery approach, which targets LECs^29,37^, to restore A20 expression in ECs of *A20*^*ΔEC*^ mice. Restoration of WT-A20 but not the DUB-inactive mutant A20 rescued VE-cad expression in lung vascular ECs of *A20*^*ΔEC*^ mice (Fig. 4c). Importantly, restoration of WT-A20 expression but not that of the DUB-inactive mutant suppressed LPS-induced lung vascular leak as measured by EBA uptake in *A20*^*ΔEC*^ mice (Fig. 4d). These findings collectively support the notion that the DUB activity of endothelial A20 is essential for maintenance and repair of the endothelial barrier following lung vascular injury.

### IRAK-M-mediated A20 expression in ECs is essential for maintenance and restoration of endothelial AJ integrity after LPS-induced endothelial injury

**Fig. 5:**
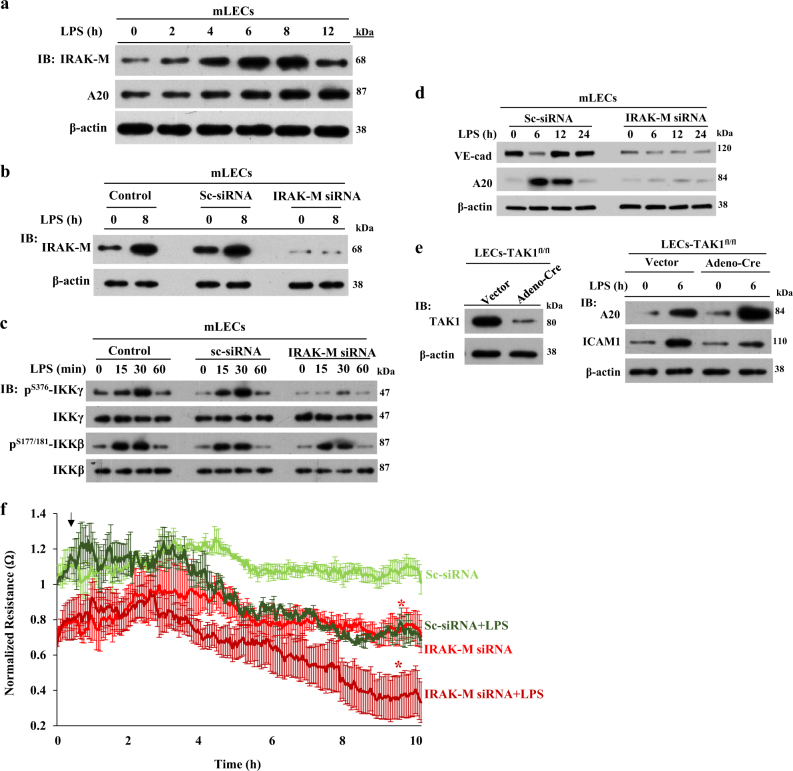
IRAK-M–IKKγ–NF-κB axis activation downstream of TLR4 induces A20 expression in ECs and signals VE-cad expression at AJs. **a** Lung endothelial cells (mLECS) from wild-type (C57BL/6) mice treated with LPS (1 µg/ml) for different time intervals were used for immunoblot to determine IRAK-M and A20 expression. **b** Control mLECs, mLECs transfected with Sc-siRNA or *m*IRAK-M-siRNA (100 nM) for 72 h were treated with LPS (1 µg/ml) and used for IB to determine IRAK-M expression. **c** Control mLECs, mLECs transfected with Sc-siRNA or *m*IRAK-M-siRNA (100 nM) for 72 h were treated with LPS (1 µg/ml) and the cell lysates were used for IB to determine phosphorylation of IKKγ (Ser376) and IKKβ (S177/181). Blots were re-probed with antibodies specific to either IKKγ or IKKβ. **d** mLECs treated with either Sc-siRNA or IRAK-M siRNA as in **b** were challenged with LPS (1 µg/ml) for different time periods and used for IB to determine VE-cad and A20 expression. **e** LECs from *TAK1* (*Map3k7*)-floxed (*TAK1*^*fl/fl*^) mice infected with (25 pfu/cell) control adenovirus (vector) or adenovirus expressing *Cre*-recombinase (Adeno-*Cre*) for 48 h were treated with or without LPS (1 µg/ml) challenge and used for IB to determine TAK1 expression (left panel) or ICAM-1 and A20 expression (right panel). **a-e** representative data from three experiments are shown. **f** HLMVECs transfected with Sc-siRNA or IRAK-M-siRNA as **b**. At 24 h after transfection, cells plated on gold electrodes were challenged with 1 µg/ml LPS in the presence of 10% FBS containing medium, and endothelial AJs integrity was monitored by TER (*n* = 4/group). *p < 0.01, compared with respective controls

**Fig. 6:**
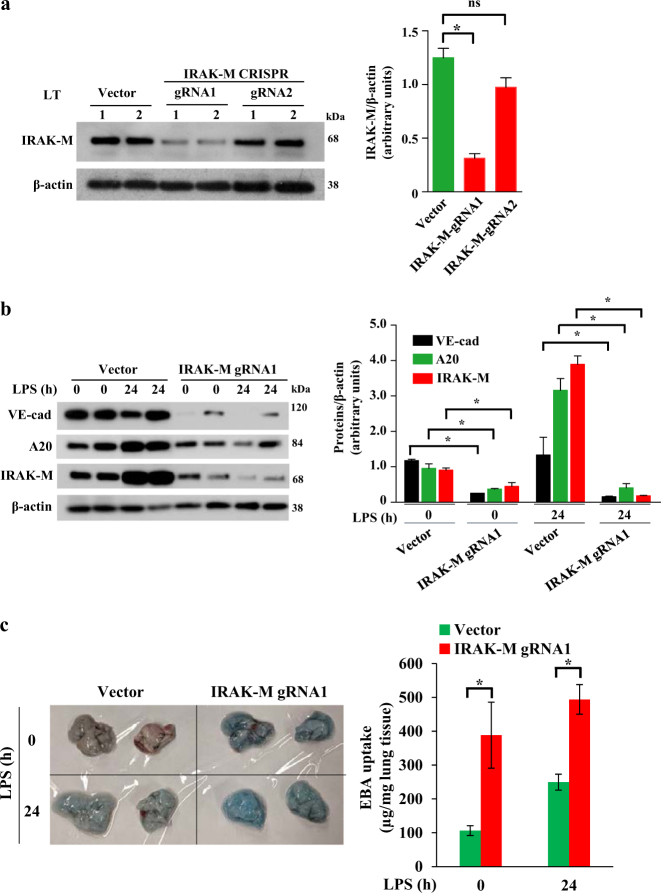
CRISPR/Cas9 system-mediated silencing of IRAK-M in mouse lung vascular ECs impaired the repair of endothelial barrier after LPS-induced lung injury. **a** WT (C57BL/6) mice were injected i.v. with liposome-CRISPR/Cas9 plasmid constructs containing two different guide RNA (gRNA1, gRNA2) against mouse IRAK-M gene. At 96 h after injection, lungs harvested were used for western analysis. *n* = 4 mice each group; *p < 0.01, vector vs gRNA1. **b**, Mice were i.v. injected with liposome-CRISPR/Cas9-IRAK-M (gRNA1) plasmid complex. At 96 h after injection, mice were challenged with LPS (5 mg/kg) for 24 h and then lungs harvested were used for immunoblot to determine expression of IRAK-M, A20, and VE-cad. *n* = 4 mice each group; *p < 0.01, vector vs gRNA1. **c** Mice injected with liposome-CRISPR/Cas9-IRAK-M (gRNA1) plasmid complex as in **b** were used to assess in vivo lung vascular leak. *n* = 4 mice per group. *p < 0.01; Vector injected mice versus IRAK-M-gRNA1 injected mice

**Fig. 7:**
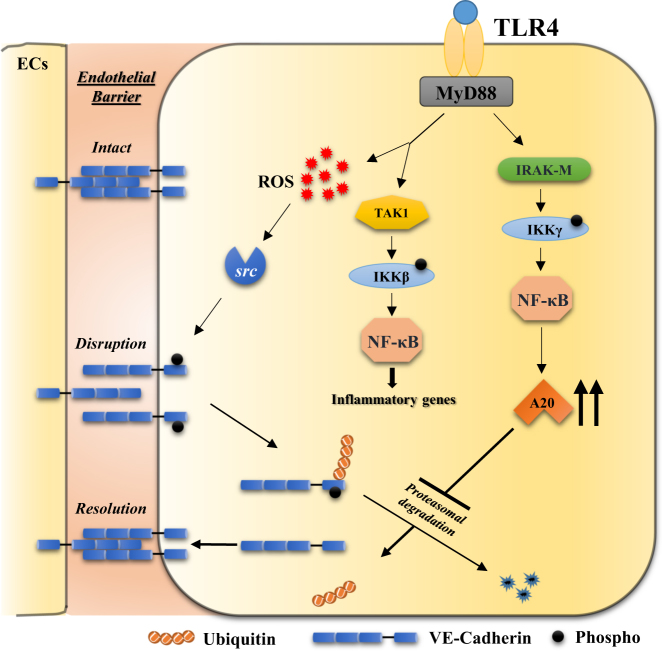
Model for TLR4-induced endothelial AJs disassembly, A20 expression in ECs, and A20-mediated repair of endothelial barrier. ROS-mediated *Src* activation downstream of TLR4 induces VE-cad phosphorylation to disassemble AJs. Subsequently, internalized phosphorylated VE-cad ubiquitinated and degraded via proteasomal pathway. A20 induced by IRAK-M–IKKγ–NF-κB axis downstream of TLR4 in ECs prevents ubiquitination of VE-cad to replenish VE-cad at AJs to restore the endothelial barrier integrity. ROS reactive oxygen species

A20 is expressed basally at a low level and its transcription is tightly regulated by NF-κB signaling^19,20^. Recent evidence suggests that two distinct NF-κB signaling pathways (i.e., TAK1-dependent and IRAK-M-dependent) are activated downstream of TLRs^38^. The TAK1-dependent pathway induces expression of pro-inflammatory genes (e.g., IL-6, TNF-α, ICAM-1), whereas the IRAK-M-dependent pathway induces anti-inflammatory genes (e.g., A20, SOCS1)^38^. The TAK1-dependent pathway mediates IKKα/IKKβ phosphorylation-dependent NF-κB activation, whereas the IRAK-M-dependent pathway triggers IKKγ phosphorylation-dependent NF-κB activation^38^. Further, IRAK-M was shown to be expressed only in myeloid cells^39,40^, but we observed IRAK-M expression in lung microvascular ECs (Fig. 5a). However, whether IRAK-M signaling in ECs is essential for A20 expression and thereby IRAK-M regulates endothelial barrier are unclear. Thus, we investigated the potential role of endothelial IRAK-M in regulating endothelial barrier integrity through A20 expression in ECs. We observed that LPS-induced both IRAK-M and A20 expression in a time-dependent manner in ECs (Fig. 5a). IRAK-M knockdown in ECs, prevented LPS-induced phosphorylation of IKKγ but not IKKβ (Figs. 5b, c). In line with these results, we observed that IRAK-M knockdown prevented both A20 and VE-cad expression in the basal state and after LPS challenge in ECs (Fig. 5d). To rule out the possible role of TAK1-dependent NF-κB signaling, we measured LPS-induced A20 expression in TAK1-deficient LECs and observed that A20 expression in response to LPS was not altered in TAK1-deficient LECs (Fig. 5e), suggesting that IRAK-M is essential for A20 induction in ECs. Next, we measured TER in control and IRAK-M-knockdown HLMVECs to assess the role of IRAK-M in regulating endothelial barrier integrity. We observed that in IRAK-M-knockdown HLMVECs, the basal value, as well as the LPS-induced decrease in TER was significantly higher than the control (Sc-siRNA treated) HLMVECs (Fig. 5f). Next, to address the in vivo role of IRAK-M in regulating endothelial barrier integrity through A20 expression, we silenced IRAK-M in mouse lung vascular ECs through liposome-mediated delivery of the CRISPR/Cas9 system^29,37^. We used two different gRNAs to target mouse (*m*) IRAK-M in the CRISPR/Cas9 system to silence IRAK-M in vivo. We observed that *m*IRAK-M-gRNA1 effectively suppressed IRAK-M in mouse lung vascular ECs (Fig. 6a). Interestingly, *m*gRNA1-mediated silencing of IRAK-M suppressed basal, as well as recovery of VE-cad expression after LPS challenge in mouse LECs (mLECs; Fig. 6b). Importantly, IRAK-M silencing in mouse lung vascular ECs prevented A20 expression (Fig. 6b). Further, we observed that basal and LPS-induced lung vascular leak was augmented in lungs of IRAK-M silenced mice compared with control mice (Fig. 6c). These results collectively support the concept that IRAK-M-mediated A20 expression in ECs is a critical determinant of endothelial barrier stability. On the basis of our key findings, we propose a model (Fig. 7) to account for LPS-induced endothelial AJs disassembly and the subsequent signaling of A20 induction that orchestrates the repair of the endothelial barrier.

## Discussion

In ALI and its most severe manifestation, acute respiratory distress syndrome, loss of VE-cad expression at endothelial AJs leads to sustained extravasation of neutrophils and monocytes and development of protein-rich pulmonary edema due to uncontrollable vascular leak^15–18,41,42^. However, little is known about the intrinsic endothelial signaling mechanisms activated to repair the endothelial barrier after vascular injury. Here, we have identified the essential role of the ubiquitin editing enzyme A20 in maintaining and repairing the endothelial barrier using a mouse model of EC-restricted A20 deletion. Mice with EC-specific A20 deficiency were viable but demonstrated enhanced basal vascular leak and inflammatory cell extravasation in lungs compared with the control mice. Importantly, *A20*^*∆EC*^ mice exhibited heightened susceptibility to LPS-induced septic shock. Further, LPS challenge resulted in uncontrollable lung vascular leak and persistent sequestration of PMNs in lungs of *A20*^*∆EC*^ mice compared with WT counterparts. In line with these observations, we observed markedly reduced VE-cad protein but not VE-cad mRNA expression in *A20*^*∆EC*^ mice. To address the specific role of A20 in regulating endothelial barrier integrity, we restored A20 expression in lung vascular ECs of *A20*^*∆EC*^ mice through liposome-mediated delivery of A20-expressing constructs. We observed that WT-A20 but not a DUB-inactive A20 expression construct restored VE-cad expression to normal levels and resolved LPS-induced lung vascular leak in *A20*^*∆EC*^ mice. Together, these results show a crucial role of A20 in regulating endothelial barrier integrity through VE-cad expression at endothelial AJs.

To address whether cultured ECs from *A20*^*∆EC*^ mice also exhibit loss of VE-cad expression, we isolated primary mLECs from *A20*^*∆EC*^ mice and observed markedly reduced VE-cad expression, resulting in failure to form normal AJs. Further, knockdown of A20 in HLMVECs also promoted loss of VE-cad expression. In response to LPS challenge, control ECs showed a time-dependent decrease in VE-cad, and VE-cad expression returned to baseline within 24 h of LPS challenge, whereas in A20 knockdown HLMVECs, VE-cad expression was not restored. Importantly, A20 deficiency decreased basal endothelial AJ stability and also increased susceptibility to LPS-induced disassembly of endothelial AJs in HLMVECs. These results establish the role of A20 in regulating endothelial barrier integrity.

As VE-cad ubiquitination may lead to loss of VE-cad expression at endothelial AJs^9^, we investigated whether A20’s DUB activity could prevent VE-cad downregulation to maintain endothelial barrier integrity. A20’s DUB domain can cleave K63-linked and K48-linked ubiquitin chains^22–24^ and was also shown to prevent E2–E3 interactions to inhibit ubiquitination of proteins in cells^25^. We co-expressed HA-tagged ubiquitin, VE-cad, and WT-A20 or DUB-inactive A20 in ECs. VE-cad immunoprecipitation and immunoblot analysis revealed that VE-cad was modified with both K63-linked and K48-linked ubiquitin chains. Interestingly, co-expression of WT-A20 but not the DUB-inactive mutant prevented ubiquitination of VE-cad, indicating either that A20 cleaved both the K63- and K48-linked ubiquitin chains from VE-cad^22–24^ or it prevented VE-cad ubiquitination by blocking E2–E3 interaction in ECs^25^. Further, to validate the in vivo role of A20, we restored A20 expression in lung vascular ECs of *A20*^*ΔEC*^ mice using the liposome-mediated gene delivery approach, which targets LECs^29,37^. Restoration of WT-A20 but not the DUB-inactive A20 mutant rescued VE-cad expression and suppressed LPS-induced lung vascular leak in *A20*^*ΔEC*^ mice. These key findings support the concept that the DUB function of endothelial A20 is essential for maintenance and repair of the endothelial barrier following lung vascular injury.

A20 expression is tightly regulated by NF-κB activation downstream of the Toll-like receptors (TLRs), the receptor for IL-1, and the receptor for TNF^19,20^. Induced A20 negatively regulates NF-κB signaling in the feedback loop to suppress inflammatory responses^26^. We showed recently that A20 expression in ECs is required to resolve lung vascular injury in mice after LPS challenge^29^. However, little is known about the crucial NF-κB signaling pathway that mediates A20 expression in ECs. Recent studies have demonstrated the co-existence of two parallel, TLR-mediated NF-κB activation pathways: TAK1-dependent and IRAK-M-dependent, respectively, in macrophages^38^. IRAK-M (also known as IRAK-3) was identified as a critical negative regulator of TLR/IL-1R family signaling via inhibition of MyD88 and IRAK1/4 activation^40^. Further, IRAK-M-deficient mice exhibited heightened susceptibility to inflammation in experimental models^39,43^. In addition, studies have demonstrated that the IRAK-M-dependent NF-κB signaling pathway is essential for the induction of anti-inflammatory genes (e.g., A20, SHIP1, SOCS1) that are not regulated at the post-transcriptional level^38^. Similar to A20 induction, LPS also induces IRAK-M expression in monocytes and macrophages; however, whether IRAK-M signaling in ECs is required for A20 expression is not known. We observed that LPS challenge induced both IRAK-M and A20 expression in a time-dependent manner in ECs. In IRAK-M-knockdown ECs, LPS failed to induce A20 expression. Importantly, VE-cad expression was also suppressed in IRAK-M-knockdown ECs, indicating that IRAK-M-dependent A20 expression promotes VE-cad expression at AJs to stabilize the endothelial barrier. To address the in vivo role of IRAK-M in regulating vascular barrier integrity, we silenced IRAK-M in mouse lung vascular ECs through liposome-mediated delivery of the CRISPR/Cas9 system. Consistent with the cultured EC results, we observed that in vivo silencing of IRAK-M in mLECs, prevented A20 expression, and suppressed basal, as well as recovery of VE-cad expression after LPS-induced lung vascular injury. Interestingly, the basal and LPS-induced lung vascular leak was augmented in lungs of IRAK-M silenced mice. These results for the first time support the role of IRAK-M-mediated A20 expression in ECs in maintaining and repairing the endothelial barrier after lung vascular injury.

In conclusion, our study reveals a novel role of endothelial A20 in regulating endothelial barrier integrity and thereby controlling vascular homeostasis. Our study demonstrates that the DUB function of endothelial A20 is required to maintain and repair the endothelial barrier after inflammatory lung vascular injury through VE-cad expression at endothelial AJs. Further, we have identified the novel function of endothelial IRAK-M in regulating endothelial barrier integrity by controlling A20 expression. Thus, our findings suggest that targeting the DUB functionality of endothelial A20 is a potential therapeutic strategy to restore endothelial barrier integrity in the setting of ALI.

## Materials and methods

### Reagents

Antibodies specific to proteins were obtained from the vendors, with catalog numbers indicated in parenthesis. Polyclonal antibody (pAb) against VE-cadherin (sc-6458) (Santa Cruz Biotechnology); pAb against VE-cadherin (ab33168) (Abcam); monoclonal Ab (mAb) against A20 (AM63-100UG) (Calbiochem); pAb against A20 (sc-32523) (Santa Cruz Biotechnology); pAb against vWF (Ab7356) (Millipore); pAb against p-TAK1^T184/187^ (4531S) (Cell Signaling Technology); pAb against TAK1 (4505S) (Cell Signaling Technology); mAb against p-P38 ^T180/Y182^ (9216S) (Cell Signaling Technology); pAb against p38 MAPK (9212 S) (Cell Signaling Technology); pAb against IKKβ (2684) (Cell Signaling Technology); pAb against p-IKKβ^S177^ (2078 S) (Cell Signaling Technology); pAb against p-IKKγ^S376^ (2689S) (Cell Signaling Technology); K63-linkage-specific polyubiquitin mAb (5621S) (Cell Signaling Technology); K48-linkage-specific polyubiquitin mAb (05-1307) (Millipore); mAb against IKKγ (05-631) (Millipore); pAb against phospho-VE-cadherin^Y731^ (44-1145G) (Invitrogen); pAb against phospho-VE-cadherin^Y685^ (ab119785) (Abcam); mAb against β-actin (A5441) (Sigma); pAb against IRAK-M (23551102) (Sigma); LPS *E. coli* 011:B4 (cat # L3013) was from Sigma (St. Louis, MO). Human WT-A20 and DUB-inactive mutant A20 (A20^C103A^) expression constructs were prepared as described^21^. Adeno-Cre-GFP (cat #1700) was from Vector Biolabs Philadelphia, PA. Scrambled-siRNA (Sc-SiRNA), human (*h*)-specific siRNA against A20 containing sequences: 5′-GCUUUCAGUUCAAGCAGAUtt-3′, 5′-AUCUGCUUGAACUGAAAGCtt-3′, 5′-CAUCCAAGGUAUACAUACAtt-3′, 5′-UGUAUGUAUACCUUGGAUGtt-3′, 5′-CAUACUCAUGGUUACUGGtt-3′, 5′-CCAGUAACCAUGAGUAUGAtt-3′; mouse (*m*)-specific siRNA against IRAK-M containing sequences: 5′-CAACGAGCUAUCCACUUAAtt-3′, 5′-UUAAGUGGAUAGCUCGUUGtt-3′, 5′-GUUCGAAUCAGCGUAUUGAtt-3′, 5′-UCAAUACGCUGAUUCGAACtt-3′, 5′-CGAGUGAGAUCUUGGUAUAtt-3′, 5′-UAUACCAAGAUCUCACUCGtt-3′; *h*-specific siRNA against IRAK-M containing sequences: 5′-GGAUGUUCGUCAUAUUGAAtt-3′, 5′-UUCAAUAUGACGAACAUCCtt-3′, 5′-CUACAGCUUUGGAAUUGUAtt-3′, 5′-UACAAUUCCAAAGCUGUAGtt-3′, 5′-CAGUUCUUCUUGUGAAGAAtt-3′, 5′-UUCUUCACAAGAAGAACUGtt-3′ obtained from Santa Cruz Biotechnology.

### Animals

*A20*^*fl/fl*^ mice were generated as described previously^32^. *TAK1*^*fl/fl*^, C57BL/6, and B6.Cg-Tg(Cdh5-Cre)7Mlia/J (VE-cadh Cre^+^) mice were obtained from Jackson Laboratories. *A20*^*fl/fl*^ mice were crossed with VE-cadh Cre^+^ (VE-cadherin promoter driving the expression of Cre) mice to generate EC-restricted *A20* knockout (*A20*^*ΔEC*^) mice as described previously^11^. All mice were housed in the University of Illinois Animal Care Facility in accordance with institutional guidelines of the US National Institutes of Health. All animal experiments were performed under the protocol approved by the Institutional Animal Care and Use Committee of the University of Illinois at Chicago.

### Lung injury assessment in mice

Age-matched *A20*^*fl/fl*^ (WT) and *A20*^*ΔEC*^ mice of either sex received indicated concentrations of a single dose of LPS (*E. coli* 011:B4) intraperitoneally^29^. For histology, 5-μm paraffin-embedded sections prepared from the lungs were stained with hematoxylin-and-eosin. For MPO assays, lungs were perfused with phosphate-buffered saline (PBS) to remove all blood and then used to measure MPO activity^29^. Cytokines in the lung tissue extracts were measured by enzyme-linked immunosorbent assay (eBioscience) following the manufacturer’s instructions. Lung microvascular permeability K_f,c_ was measured using isolated lung preparations as described^44^. In vivo mouse lung vascular leak (permeability) was assessed by measuring uptake of EBA as described^11^. Thirty minutes after EBA injection (20 mg/kg), mice were euthanized, and lungs harvested were used to measure EBA concentration^11^.

### Cell culture

HLMVECs and endothelial growth media-2 (EGM-2) were purchased from Lonza (Walkersville, MD, USA). mLECs were isolated and cultured as described by us^11,44^. HLMVECs were cultured in EGM-2MV supplemented with 15% fetal bovine serum (FBS), and mLECs were grown in EGM-2 supplemented with 5% FBS. Both cell types were used between passages 3 and 6. HMEC was grown in endothelial basal medium MCDB131 supplemented with 10% FBS, 10 ng/ml epidermal growth factor, 2 mM l-glutamine, and 1 µg/ml hydrocortisone^36^. BMDMs from mice were generated by culture of bone marrow cells as described previously^28^.

### Immunostaining

Confluent ECs grown on glass coverslips were washed quickly with ice-cold PBS and fixed with 2% paraformaldehyde (PFA) for 15 min at 4 °C. Following fixation, cells were permeabilized with 0.05% Triton X-100 for 1 min at 4 °C. Next, cells were washed three times with PBS and then incubated with blocking buffer (PBS containing 5% horse serum and 1% bovine serum albumin (BSA)) for 1 h, at room temperature (RT). Cells were then incubated overnight with the indicated primary antibody (in PBS containing 1% BSA) at 4 °C. The next day, cells were washed three times and incubated with specific Alexa-Fluor-conjugated secondary antibody and 4',6-diamidino-2-phenylindole (DAPI) for 1 h at 4 °C. Finally, cells were washed three times and mounted on glass slides for viewing. Images were acquired with the Zeiss LSM 510 confocal microscope.

Lung tissue sections were immunostained as described^45^. Briefly paraffin-embedded formalin-fixed sections were deparaffinized by passing through a xylene and ethanol series followed by rehydration in dH_2_O. The antigen retrieval was done by boiling the slides in antigen retrieval buffer (10 mM citrate, 0.05% Tween 20, pH 6.0) for 20 min. Then, the sections were incubated in blocking buffer (PBS containing 4% BSA, 0.2% Triton X-100) for 2 h at RT. The sections were incubated with antibodies specific to A20 and von Willebrand factor (endothelial marker) overnight in 0.5% BSA blocking buffer and staining was detected by incubation with the appropriate fluorochrome coupled secondary antibody for 1 h at RT. The sections were mounted with DAPI supplemented anti-fade mounting solution (Invitrogen) and the images were acquired with the Zeiss LSM 510 confocal microscope.

### Immunoprecipitation and immunoblotting

Cells were washed three times with PBS at 4 °C and lysed in lysis buffer (50 mM Tris-HCl, pH7.5, 150 mM NaCl, 1 mM EGTA, 1% Triton X-100, 0.25% sodium deoxycholate, 0.1% sodium dodecyl sulfate (SDS), 10 μM orthovanadate, and protease-inhibitor mixture) as previously described^46^. Mouse lungs were homogenized in lysis buffer^29^. Cell lysates were centrifuged (13,000 × *g* for 10 min) to remove insoluble materials. Clear supernatant (300 μg protein) was subjected to immunoprecipitation. Each sample was incubated overnight with 1 μg/ml of the indicated antibody at 4 °C. The next day, protein A/G beads were added to the sample and incubated for 1 h at 4 °C. Immunoprecipitates were then washed three times with wash buffer (Tris-buffered saline containing 0.05% Triton X-100, 1 mM Na_3_VO_4_, 1 mM NaF, 2 μg/ml leupeptin, 2 μg/ml pepstatin A, 2 μg/ml aprotinin, and 44 μg/ml phenylmethylsulfonyl fluoride). Cell lysates, lung tissue homogenates, or immunoprecipitated proteins were resolved by SDS-polyacrylamide gel electrophoresis (PAGE) on a 4–15% gradient separating gel under reducing conditions and transferred to a Duralose membrane. Membranes were blocked with 5% dry milk in Tris-buffered saline containing 0.1% Tween-20 (TBST) (10 mM Tris-HCl pH7.5, 150 mM NaCl, and 0.05% Tween-20) for 1 h at RT and then incubated with the indicated primary antibody diluted in blocking buffer overnight at 4 °C. For phospho-specific blots, the membranes were incubated overnight at 4 °C with the primary antibody diluted in TBST containing 5% bovine serum albumin. Next, membranes were washed three times and then incubated with appropriate horseradish peroxidase-conjugated secondary antibody. Protein bands were detected by enhanced chemiluminescence. Immunoblot protein bands were quantified using NIH ImageJ software.

### siRNA transfection

ECs grown to 70–80% confluence on gelatin-coated culture dishes were transfected with target siRNAs or Sc-siRNA as described^11^. At 72 h after transfection, cells were used for experiments.

### Transendothelial electrical resistance measurements

Real-time changes in TER were measured to assess endothelial barrier function^47^. TER values of each monolayer were normalized to control monolayer (Sc-siRNA treated) baseline values.

### VE-cad ubiquitination

HMEC grown on 35 mm culture dishes were co-transfected with HA-tagged ubiquitin-expressing plasmid (1 µg/ml) and with either A20^WT^ or A20^C103A^ plasmid (2 µg/ml). At 24 h after transfection, cells were infected with recombinant adenovirus (25 pfu/cell) expressing VE-cad (Adeno-VE-cad). At 72 h, cell lysates were immunoprecipitated (IPed) with anti-VE-cad Ab. Prior to lysis, cells were pretreated with MG132 (10 µM) for 2 h. IPed proteins were boiled in sample buffer (4% SDS, 20% glycerol, 0.2% bromophenol blue, 100 mM Tris pH 6.8 and 200 mM DTT), resolved using 6% separating gel SDS-PAGE, and blotted with indicated antibodies.

### Liposome-mediated transduction of cDNA into mouse lung vascular ECs

Liposomes were prepared as described previously^29,37^. Briefly, the mixture comprised of dimethyldioctadecylammonium bromide and cholesterol (1:1 molar ratio) was dried using the Rotavaporator (Brinkmann), and dissolved in 5% glucose followed by 20-min sonication. The complex consisting of plasmid DNA-expressing WT-A20 or DUB-inactive mutant A20 (A20^C103A^) and liposomes were combined at a ratio of 1 μg of DNA to 8 nmol of liposomes. The DNA/liposome complex (25 μg of DNA/mouse) was injected into the retro-orbital venous plexus^29,37^. Forty-eight hours after injection, mice were used for experiments.

### In vivo disruption of IRAK-M gene in lung vascular ECs

CRISPR/Cas9 system (pSpCas9 BB-2A-GFP PX458) originally developed by Zhang laboratory^48^, was used to inactivate the IRAK-M gene in vivo. This all-in-one CRISPR plasmid DNA expresses Cas9 under the control of the CBh promoter and gRNA driven by the *U6* promoter^48^. The CRISPR/Cas9 plasmid containing the guide RNA sequence (gRNA1, 5′-CGGAGCGACTTTCAAACAGC-3′ or gRNA2, 5′-GATATTCGAAGTATACAGAG-3′) was custom prepared by GenScript. CRISPR/Cas9 plasmid was delivered in mouse lungs using cationic liposomes as described above. The liposomes and CRISPR plasmid mixture was injected i.v. into the mouse (30 μg of plasmid in 100 μl of liposome suspension/per mouse). Four days after injection, animals were used for experiments.

### Statistical analysis

Analysis of variance and Student’s *t-*test (two-tailed) were used to determine statistical significance with a *p*-value threshold set at < 0.05.
